# Establishment of normal myofiber size distribution in children and young adults

**DOI:** 10.1093/jnen/nlaf123

**Published:** 2025-11-04

**Authors:** Michael W Lawlor, Marta Margeta, Karra A Jones, Benedikt Schoser, Jennifer A Cotter, Veena Rajaram, Steven A Moore, Mariah J Prom, Margaret Beatka, Emily Ott, Rebecca A Slick, Michael P Collins, Ann Esselman, Nazima Shahnoor, Susan Danielson, Hui Meng, Fatbardha Varfaj, Suyash Prasad, Salvador Rico, Jun Lee, Suresh N Kumar, Heather Gordish Dressman

**Affiliations:** Department of Pathology, Medical College of Wisconsin, Milwaukee, WI, United States; Diverge Translational Science Laboratory, Milwaukee, WI, United States; Department of Pathology, University of California San Francisco, San Francisco, CA, United States; Department of Pathology, Duke University School of Medicine, Durham, NC, United States; Department of Neurology, Friedrich-Baur-Institute, Ludwig-Maximilians-University Munich, Munich, Germany; Children’s Hospital Los Angeles, Keck School of Medicine of University of Southern California, Los Angeles, CA, United States; Department of Pathology, UT Southwestern Medical Center. Dallas, TX, United States; Carver College of Medicine, The University of Iowa, Iowa City, IA, United States; Diverge Translational Science Laboratory, Milwaukee, WI, United States; Diverge Translational Science Laboratory, Milwaukee, WI, United States; Diverge Translational Science Laboratory, Milwaukee, WI, United States; Department of Pathology, Medical College of Wisconsin, Milwaukee, WI, United States; Department of Pathology, Medical College of Wisconsin, Milwaukee, WI, United States; Department of Neurology, Medical College of Wisconsin, Milwaukee, WI, United States; Department of Pathology, Medical College of Wisconsin, Milwaukee, WI, United States; Department of Pathology, Medical College of Wisconsin, Milwaukee, WI, United States; Department of Pathology, Medical College of Wisconsin, Milwaukee, WI, United States; Diverge Translational Science Laboratory, Milwaukee, WI, United States; Formerly of Astellas Gene Therapies (formerly Audentes Therapeutics), San Francisco, CA, United States; Formerly of Astellas Gene Therapies (formerly Audentes Therapeutics), San Francisco, CA, United States; Formerly of Astellas Gene Therapies (formerly Audentes Therapeutics), San Francisco, CA, United States; Formerly of Astellas Gene Therapies (formerly Audentes Therapeutics), San Francisco, CA, United States; Department of Pathology, Medical College of Wisconsin, Milwaukee, WI, United States; Children’s National Hospital and the George Washington University, Washington, DC, United States

**Keywords:** fiber size, growth curve, minFeret diameter, muscle biopsy, muscle growth, muscle pathology

## Abstract

Abnormalities of myofiber size are often of diagnostic significance on skeletal muscle biopsies, mainly when myofibers are excessively small. The establishment of standards for myofiber size in children has been hampered until recently by the lack of tools to assess large numbers of fibers across a sizeable number of samples. This study describes the assessment of myofiber size in 349 histologically normal patient biopsy specimens obtained between 4 weeks and 25 years of life and corresponding primarily to locations in the thigh/quadriceps/vastus lateralis region. Biopsy specimens were selected for inclusion based on histologically normal light microscopic findings and minimal technical artifacts. H&E-stained slides were scanned and evaluated for minFeret diameter using a Visiopharm software app (APP #10164). MinFeret diameter fiber size data were then grouped into 18 age cohorts to establish normal ranges for males and females within each age cohort. A pilot study to compare known abnormal cases to these normal ranges was then performed to demonstrate how cases with abnormal fiber size might compare to these standards. This dataset provides a user-friendly and applicable set of standard fiber size ranges to assist in diagnostic and scientific work in children and young adults.

## INTRODUCTION

The size of skeletal muscle fibers (myofibers) can be an important indicator of muscle health and growth, reflecting the number of myofilaments available to conduct muscle contractions. Myofiber size increases with age throughout childhood but this relationship has not been well studied historically due to technical limitations. Muscle biopsy specimens can contain thousands of myofibers, and until recently, the methods for myofiber size measurement required manual measuring and counting of each fiber. With emerging improvements in image analysis software and automation, it is now possible to perform more comprehensive studies of myofiber size using large numbers of patients across a wide range of ages in children and young adults.

Establishing normal ranges for myofiber size enables more specific assessments of myofiber size abnormalities; this will likely be helpful for diagnostic skeletal muscle pathology and clinical trials. Myofiber smallness is a key pathological feature in some primary muscle diseases (including many of the congenital myopathies), but recognizing myofiber smallness often necessitates the presence of a larger fiber population to demonstrate the potential for more fiber growth in that patient. The recognition of fiber size abnormalities in biopsy samples in which all fibers are small, however, can be complex without well-defined standards for a given age. Additionally, some emerging experimental therapies can improve disease-associated abnormalities of myofiber size. This makes myofiber size a potentially important endpoint as these therapies progress through human clinical studies. The establishment of better-defined standards for fiber size with age will be useful for defining the likely ceiling of myofiber size recovery at a given age and for determining whether fiber size increases are associated with a treatment-related versus age-related cause when assessing serial biopsies in studies that span multiple years.

Several prior studies have evaluated fiber size in focused patient populations. Still, it has only recently become possible to generate fiber size data on the many patient specimens required to establish standards related to myofiber size in growing patients. Arguably, the most commonly used pediatric myofiber size standards were established by a single study from the 1960s in which patterns of fiber size variation were evaluated in 180 biopsy samples from patients under age 15 years (with just 42 of these biopsies appearing histologically normal)^1^. Subsequent studies of additional pediatric and adult autopsy material,^2^^,^^3^ adults between ages 18-34,^4^ and males and females at 16 years of age^5^ provided further data. More recently, a systematic survey of myofiber size and type was performed using biopsy samples from 55 women and 95 men at approximately 21 years of age; this likely provides an excellent set of standards for fiber size in young adults.^6^ The broadest characterization of fiber size and type corresponds to a study of 83 biopsies from the deltoid muscle obtained from patients between birth and 18 years of age, using computerized image analysis tools similar to those implemented in this study.^7^

The current study extends this work further by analyzing 349 scanned hematoxylin and eosin (H&E)-stained slides from histologically normal muscle biopsy specimens that were selected from the historical case archives of several large institutions. Cases were selected retrospectively based on: (1) the lack of pathological findings on their initial muscle biopsy reports and (2) confirmation that histological abnormalities were not present when the key pathology review authors (all experienced pediatric neuromuscular pathologists) reviewed the H&E-stained slides of these cases. The slides were analyzed using recently developed computational assessments (with manual quality control) and statistical analysis. The sample size was sufficiently large to establish sex-specific standard fiber size ranges for relevant age groups between birth and age 25 and to establish growth curves that depict the myofiber growth in male and female individuals across this age range.

## METHODS

### Human subjects research compliance

Procedures followed were in accordance with the ethical standards of the responsible committees on human experimentation (institutional or regional) and with the Helsinki Declaration (1964, amended most recently in 2008) of the World Medical Association. All study activities, including activities at Medical College of Wisconsin and other hospitals contributing cases (University of California San Francisco, University of Texas Southwestern, University of Iowa, and University of Southern California) were performed under an exemption from IRB oversight granted by Institutional Review Board of the Medical College of Wisconsin (project ID PRO00035847, granted exemption on September 13, 2019). This exemption was considered “Exempt Category 4,” as the study involved the secondary use of identifiable private information or identifiable biospecimens, and access to personal information was limited and did not allow identification of specific patients. A waiver of consent was granted on the basis that the study involved a retrospective review of slides in a clinical laboratory information system (often from a number of years ago), which made obtaining additional consent impractical. Only retrospective cases and records were used, and access to patient information and records was limited to specifically trained study staff until appropriate cases were selected. After cases were selected (which was prior to analysis by the study team), no further patient information was accessed.

### Study design

Scanned images of H&E-stained muscle biopsy cryosections were obtained from participating institutions (Medical College of Wisconsin, University of California San Francisco, University of Iowa, Children’s Hospital Los Angeles, and University of Texas Southwestern). Criteria for including a case included: (1) patient age between birth and 25 years of age, (2) normal histological findings on institution-specific standard muscle biopsy workup and on H&E by light microscopy, and (3) high-quality sectioning and staining. Samples appropriate for use were deidentified after recording the patient’s age, sex, and muscle biopsy site and sent to the Children’s Hospital of Wisconsin Research Institute’s (CRI) Imaging Core Facility for further image analysis. The enrollment goal was to obtain up to 25 cases in each of 18 age groups (0-3 months, 4-6 months, 7-9 months, 10-12 months, 1 year, 2 years, 3 years, 4 years, 5 years, 6 years, 7 years, 8 years, 9 years, 10-12 years, 13-15 years, 16-18 years, 19-21 years, and 22-25 years). These age groupings were designed to capture specific periods in which muscle size is expected to change rapidly (such as infancy), while combining later time points across several years as muscle fiber size is expected to be more static and muscle biopsies are much less common. It was necessary to define the age groups prior to receiving IRB approval for the retrospective review, so the decisions on specific groups were based on the experience of the four diagnosticians involved in the earliest stages of the study (Drs. Lawlor, Margeta, Schoser, and Jones). There was no effort to exclude samples based on clinical history or suspected disease, which was not consistently available. It was also not possible to identify whether individual biopsies were open biopsies or core biopsies (although by their size, nearly all biopsies were too large to be core biopsies). Enrollment information is provided in Table 1.

**Table 1.** Characteristics of cases included in this study.

	Biopsies from female patients		Biopsies from male patients		Total
Age group	N	Biopsy location^a^ (n)	N	Biopsy location^a^ (n)	N^b^
0-3 months	9	Quadriceps (4); Rectus (1); Thigh (3); NOS (1)	12	Biceps (1); Quadriceps (4); Rectus (1); Thigh (4) NOS (1); Vastus lateralis (1)	21
4-6 months	5	Quadriceps (4); Rectus (1)	9	Quadriceps (1); NOS (8)	14
7-9 months	6	Quadriceps (6)	7	Quadriceps (1); NOS (5); Thigh (1)	13
10-12 months	6	Quadriceps (1); Unknown (4); Vastus lateralis (1)	1	NOS (1)	7
1 year	11	Paraspinous (1); Quadriceps (8); Thigh (1); Vastus lateralis (1)	14	Leg (1); Quadriceps (11); Vastus lateralis (2)	25
2 years	13	NOS (2); Quadriceps (5); Thigh (1); Vastus lateralis (5)	11	Quadriceps (7); Thigh (2); Vastus lateralis (2)	24
3 years	14	Deltoid (1); Gastrocnemius (1); Quadriceps (5); Thigh (1); Vastus lateralis (6)	11	Gastrocnemius (1); Quadriceps (5); Thigh (1); NOS (1); Vastus lateralis (3)	25
4 years	9	Gastrocnemius (1); Quadriceps (3); NOS (4); Vastus lateralis (1)	10	Quadriceps (6); Thigh (1); Triceps (1); NOS (2)	19
5 years	5	Quadriceps (2); NOS (3)	12	Biceps (1); Foot (1); Quadriceps (5); Thigh (1); NOS (1); Vastus lateralis (3)	17
6 years	9	Deltoid (1); Gastrocnemius (1); Quadriceps (5); NOS (2)	7	Gastrocnemius (1); Quadriceps (2); NOS (3); Vastus lateralis (1)	16
7 years	3	Quadriceps (2); NOS (1)	8	Leg (1); Quadriceps (2); Thigh (1); NOS (3); Vastus lateralis (1)	11
8 years	5	Quadriceps (2); Thigh (1); NOS (2)	9	Quadriceps (5); Thigh (1); NOS (2); Vastus lateralis (1)	14
9 years	9	Quadriceps (5); NOS (4)	6	Deltoid (1); Leg (1); Quadriceps (3); Thigh (1)	15
10-12 years	11	Deltoid (2); NOS (1); Quadriceps (2); Rectus (1); Soleus (1); Thigh (1); Vastus lateralis (3)	15	Biceps (2); Gastrocnemius (2); Quadriceps (9); Rectus (1); Vastus lateralis (1)	26
13-15 years	11	Gastrocnemius (1); Quadriceps (5); Soleus (1); NOS (1); Vastus lateralis (3)	12	Arm (1); Quadriceps (10); Vastus lateralis (1)	23
16-18 years	15	Biceps (4); Leg (2); Quadriceps (6); Thigh (2); Vastus lateralis (1)	19	Biceps (3); Deltoid (1); Quadriceps (9); Thigh (2); Vastus lateralis (4)	34
19-21 years	12	Biceps (2); Gastrocnemius (1); Quadriceps (3); Thigh (4); Vastus lateralis (2)	10	Deltoid (1); Quadriceps (6); Thigh (2); Vastus lateralis (1)	22
22-25 years	12	Biceps (4); Deltoid (3); Gastrocnemius (1); NOS (1); Quadriceps (2); Triceps (1)	11	Biceps (2); Deltoid (3); Quadriceps (5); Thigh (1)	23

a For biopsy location, note that quadriceps, vastus lateralis, and thigh likely all correspond to the same site.

b Total number of muscle biopsy samples per age group.

Abbreviations: n, number of samples by biopsy location; N, total number of biopsy samples per group; NOS, not otherwise specified.

### Image analysis, automated stage

Scanned slide images were analyzed for myofiber size with Visiopharm image analysis software (Visiopharm; Hoersholm, Denmark; version 2019.2) using APP #10164, “H&E, Centronuclear Myopathy.” This app was designed in collaboration with our laboratory to apply the Visiopharm myofiber size analysis system to H&E slides and to optimize performance across the range of fiber sizes seen from birth to adulthood, and in the context of congenital myopathies. Automated quantification of fiber size requires the accurate delineation of myofiber edges as a first step. To determine whether the Visiopharm algorithm performed appropriate myofiber recognition and myofiber segmentation in relation to the original image, the mask of cell edge recognition detected by Visiopharm was compared to areas of interest from the original image. Visual inspection by a pathologist (MWL) was used to determine whether the software appropriately recognized myofibers, and a specimen was excluded from analysis if recognition of fibers was poor. The failure to recognize a small number of fibers appropriately was acceptable if those fibers were within the fiber size range of recognized fibers and if the vast majority (>95% of fibers) were identified within the image or slide scan. The segmentation protocol was sufficiently broad to capture fibers for 50 to 50 000 μm^2^ area. Following automated tissue detection to identify the specimen, the specimens were analyzed at 40× magnification by the fiber type app that used preset identifiers to segment fibers from interstitial tissues and artifacts. Only 50-75% of each field scanned was analyzed using a counting frame to maintain fiber size integrity and to avoid double counting of the fibers. The fiber measurements including area and minimum diameter were automatically generated by the app as csv files that were further analyzed by Excel software (Supplementary Material: Standard Operating Procedure for Automated Quantification Utilizing GraphPad and Excel). Data from the masked images were manually verified for accuracy by ensuring that cell edges detected by the software corresponded to visually recognizable myofiber edges.

### Image analysis, manual stage

For determining thresholds related to myofiber size, the best agreement between manual and automated quantification was seen when manually measured minimum and maximum fiber size thresholds were applied to the computerized dataset. After quantification masks were reviewed to confirm appropriate myofiber recognition, thresholds for realistic minimum and maximum values for the sample were applied to exclude measurements that were either too large or too small to correspond to myofibers in the specimen. In this way, the datasets excluded small and large areas that are appropriate in shape but do not correspond to myofibers (such as capillaries or spaces between fibers). These minimum and maximum thresholds were identified by manually measuring the largest and smallest fibers in a subset of samples from each age group and are shown in Table S1. Following the exclusion of measurements outside the defined fiber size range, the data were used for graphing and statistical analyses, as described below.

### Construction of cumulative probability and frequency histograms

Cumulative probability plots and frequency histograms were made in R statistical computing software (version 2022.07.1), which can be downloaded at http://www.r-project.org/ (accessed 24FEB2022). Programming details are available in the Supplementary Material (Standard Operating Procedure for Automated Quantification Utilizing R Programming Language). Excel spreadsheets containing fiber size data were loaded into RStudio and muscle fiber measurements outside of ranges for a given age (Table S1) were filtered out of each dataset. Data were then cleaned and sorted from minimum to maximum fiber size diameter using tidyr and dplyr. Cumulative sums and probabilities were calculated for each measurement using base R functions (“cumsum,” “tail,” and “sum”). Cumulative probability plots and frequency histograms were generated using the ggplot2 package. Bins of 5 were used for frequency histograms to plot the count of each fiber measurement over the sum of each count. When evaluating cumulative probability plots, a single line is shown for each sample, with fiber size plotted on the X-axis and the probability that a fiber will be at or less than that fiber size plotted at the Y axis. This allows a representation of the data where an approximately sigmoid curve is plotted for each sample and where the slope of different areas of the curve reflects the distribution of fiber sizes over a range of interest. Samples with minimal variation in fiber size will have a very steep slope, and symmetrical growth over time would be seen as a gradual rightward shift of the entire curve. In cases in which there is significant variation in fiber size, there will be variation in the slope of the curve to reflect the overall dispersion of fiber sizes and to illustrate where values are clustered.

### Qualification/validation of Visiopharm measurements

Six slide scans from H&E-stained slides were selected from our files with the intention of including myofiber sizes across the range observed in our clinical trial work. For this assessment, screenshots were generated at 200× magnification (ie, 20× objective) on a 27-inch computer monitor. For manual fiber size measurements, measurements were made by a laboratory technician, using ImageJ software (Fiji (ImageJ) Version 2.0.0-rc-49/1.51d—Build: e01a259e5d) to make point-to-point assessments and a visual approximation of the most appropriate angle of measurement for the determination of minFeret diameter in each fiber. Because the Visiopharm software could not evaluate screenshot images, it was necessary to go back to the original slide scans and select regions of interest that corresponded very closely to the areas sampled in the manually counted screenshots. These regions of interest were analyzed for minFeret diameter measurements using Visiopharm APP #10164, as described above. Once measurements were complete, values were compared across each method to determine whether manual and automated minFeret diameter measurements yielded similar results. Given that the regions of interest did not precisely correspond to the manually evaluated area and the potential for error on both the manual and automated quantification elements of the analysis, an arbitrary level of acceptable similarity was set at 15%. It should also be noted that screenshots with small numbers of myofibers are likely to amplify the differences observed compared to evaluating large, scanned areas that include much larger numbers of myofibers.

### Other statistical analyses

To provide estimates of expected fiber sizes in the normal population given the observed biopsies, mixed effects generalized linear regression models were used. In each model, fiber size was the dependent variable, age at biopsy was the independent variable, and patient was included as a random effect. These models allowed the use of all fiber size assessments per patient and account for the dependence of the assessments rather than using only summary values. Most models were performed separately in males and females; however, when testing for a difference between sexes at each age and each percentile, sex was included in the model specific to that percentile and post-hoc contrasts between sexes were performed. Consistent with child growth curves, confidence interval error bars are not shown for better readability.

## RESULTS

### Validation of myofiber size measurements

**Figure 1.**
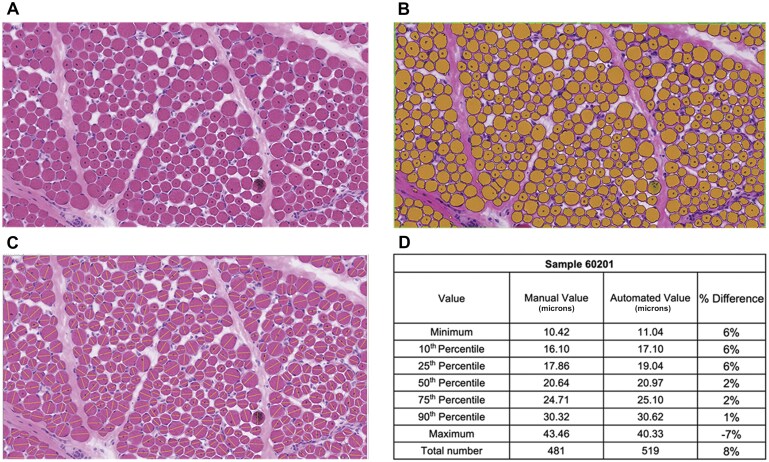
Validation of automated fiber size measurements. The quality of automated fiber size assessment data was evaluated by comparing manual versus automated fiber size (minFeret diameter) measurements within a region of interest from six samples. These biopsy specimens were known to be abnormal but were considered helpful in evaluating the technique across a broad range of fiber sizes. (A) The original screenshot from one sample was taken at 200× magnification. (B) A region of interest was defined for analysis using the automated Visiopharm platform; the segmentation between individual fibers is illustrated. The yellow area indicates areas identified as myofibers to be measured. An automated quantification dataset for the region of interest was obtained. (C) The original area of interest was evaluated in parallel using manual assessments. The axes and lengths measured are shown as yellow lines for each fiber. Note that possible errors in manual measurement include assigning an axis for minFeret diameter and any inaccuracies in drawing the line manually. (D) A comparison of automated and manual quantification values using this sample. Comparisons for all validation samples are shown in Table 2.

The quality of the automated analysis data was assessed through a validation effort that compared manual and automated measurements of the same areas of interest from six different muscle biopsy samples. The biopsy samples for this assessment were taken from X-linked myotubular myopathy (XLMTM) patients participating in the ASPIRO gene therapy trial (NCT03199469),^8^^,^^9^ as this approach was developed out of a need for accurate fiber size assessment in that study. This collection of samples included a very high variation in fiber size across samples encompassing most of the pediatric fiber size range (Figure 1). For fiber size measurement, minFeret diameter (the diameter across the minor axis on an elliptical shape) was used, as this is the fiber size measurement that is least dependent on fiber orientation. A comparison of measurements and fiber size percentiles is shown in Table 2. Overall, the goal of the assessment was to be within 15% for each percentile number in each sample, which was achieved for all percentiles in four of six patient images (60201, 9758, 27167, and 44113). The remaining two images (65481 and 25295) had excessive variation at the lowest percentiles, reflecting a subset of small areas counted as fibers by Visiopharm that did not correspond to myofibers on manual assessment or on re-review of the images after the data had been analyzed. These findings suggest a limit to the accuracy of these automated fiber size measurements when dealing with very small fiber sizes (such as less than 5 microns), but are less likely to be relevant when using whole slide scans that include thousands or tens of thousands of real fibers.

**Table 2.** Comparisons between manual and automated measurements during validation of the automated measurement approach.

	60201			65481			9758			25295			27167			44113		
Percentile	Manual	Auto	% Diff	Manual	Auto	% Diff	Manual	Auto	% Diff	Manual	Auto	% Diff	Manual	Auto	% Diff	Manual	Auto	% Diff
Minimum	10.4	11.0	5.8%	6.2	6.5	4.1%	3.1	3.1	0.6%	6.8	6.8	0.3%	11.4	12.1	5.7%	4.0	4.1	2.5%
10th	16.1	17.1	6.0%	15.4	10.9	−34.3%	6.4	6.5	1.7%	12.9	9.8	−27.7%	17.4	17.5	0.8%	7.6	8.3	8.9%
25th	17.9	19.0	6.4%	18.1	16.5	−9.2%	8.1	8.4	4.1%	15.6	13.0	−18.1%	19.6	19.3	−1.7%	8.9	9.6	7.3%
50th	20.6	21.0	1.6%	21.8	20.5	−6.1%	10.8	10.5	−2.7%	18.4	16.8	−8.7%	21.8	21.3	−2.5%	10.5	11.0	4.0%
75th	24.7	25.1	1.6%	28.6	25.5	−11.3%	19.6	17.0	−14.2%	22.0	19.8	−10.5%	24.8	23.8	−4.5%	15.5	16.8	8.4%
90th	30.3	30.6	1.0%	36.0	32.1	−11.6%	28.4	26.9	−5.3%	26.5	23.4	−12.4%	29.1	27.3	−6.4%	20.7	21.4	3.0%
Maximum	43.5	40.3	−7.5%	53.8	52.9	−1.8%	55.8	55.7	−0.2%	40.2	37.6	−6.7%	42.0	40.1	−4.6%	34.5	31.0	−10.6%
Total number	481.0	519.0	7.6%	357.0	431.0	18.8%	760.0	950.0	22.2%	493.0	572.0	14.8%	398.0	431.0	8.0%	929.0	874.0	−6.1%

Abbreviations: auto, automated; diff, difference.

### Normal fiber size ranges by age

**Figure 2.**
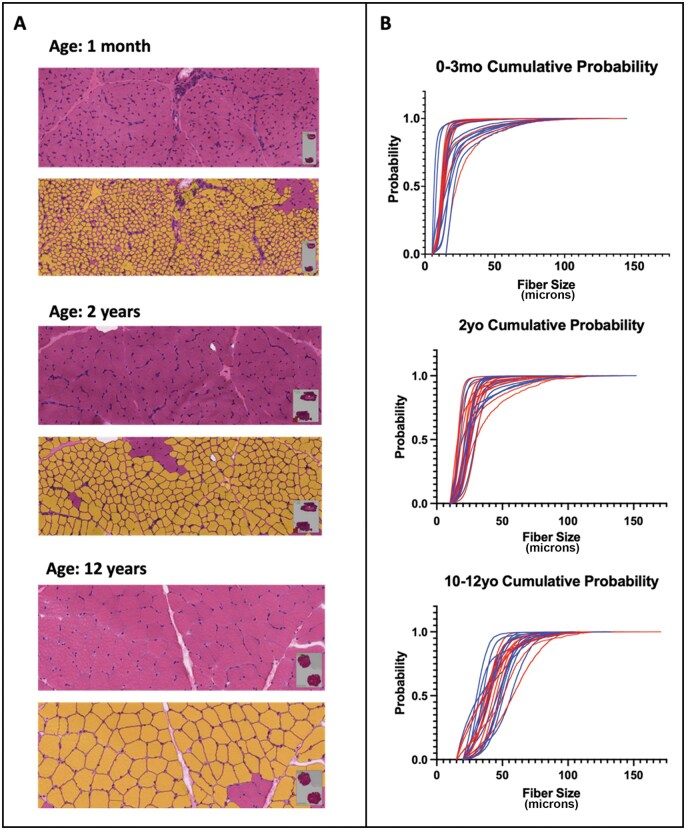
Fiber size growth across three age groups. Representative areas and cumulative probability plots to illustrate fiber size increase with age. (A) Screenshots from H&E slide scans (top) and segmentation masks (bottom) from representative samples in three different age groups. These masks illustrate issues with segmentation in the most problematic areas across an entire slide scan but the data are obtained from all fibers in the tissue section captured on the slide scan. (B) Cumulative probability plots illustrate the distribution of fiber size (minFeret diameter) values across all patients within three patient age cohorts. Each line corresponds to the fiber size data from an individual patient; blue lines correspond to males and red lines correspond to females. Rightward shifts in the cumulative probability plots indicate larger fiber sizes. Abbreviations: H&E, hematoxylin and eosin; mo, months old; yo, years old.

**Figure 3.**
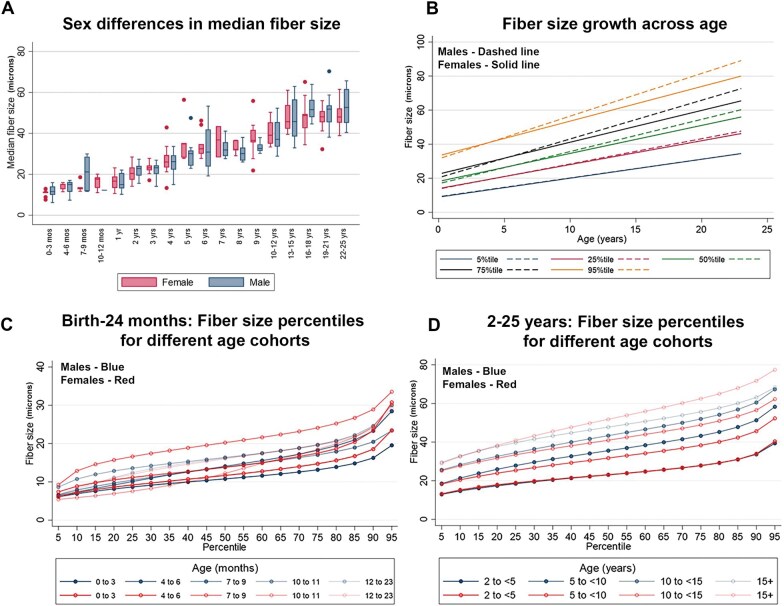
Distributions of fiber size by sex and age group. (A) Sex differences in myofiber size (minFeret diameter) across age groups: box plot depicting the median (horizonal line), interquartile range (box), and minimum-maximum range (whiskers) of median fiber sizes per sample, reported by sex within each age group. (B) Assessment of age-related fiber size changes in select fiber size percentiles (5^th^, 25^th^, 50^th^, 75^th^, and 95^th^), separated by sex (males, dashed lines; females, solid lines). (C, D) Comparisons between fiber size and age in males (blue) and females (red) are shown for patients 2 years old or younger (C) and patients between 2 and 15+ years of age (D).

**Figure 4.**
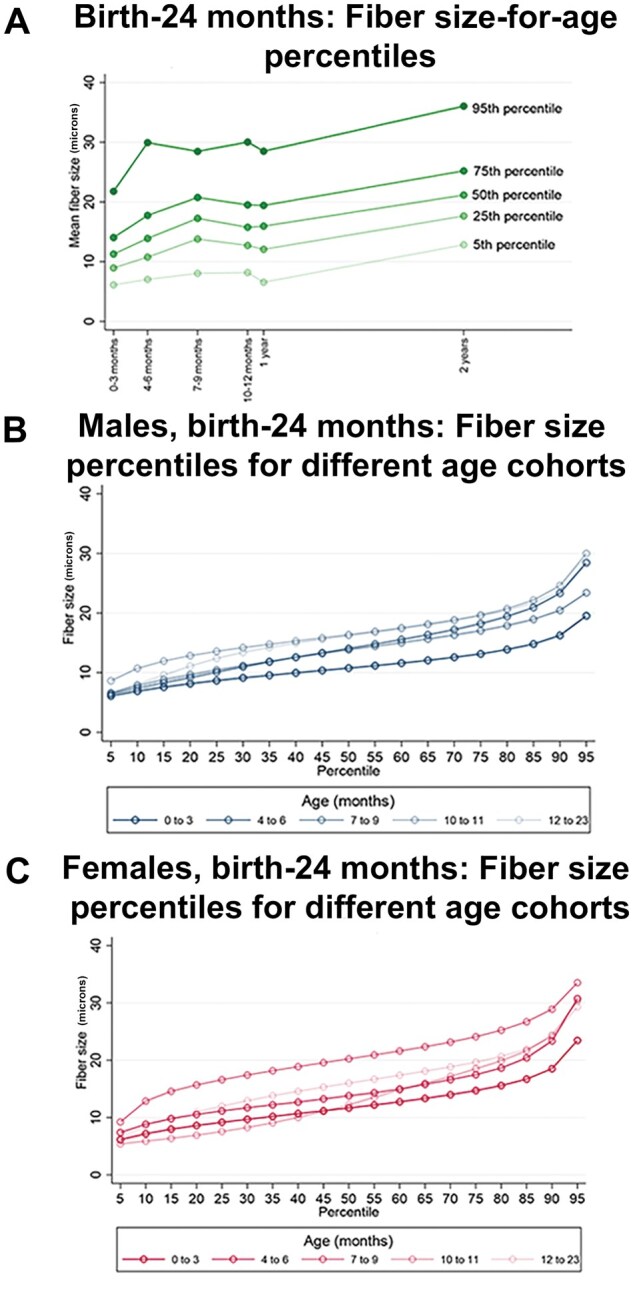
Fiber size growth from birth to 2 years of age. (A) Assessment of fiber size (minFeret diameter) at select percentiles (5^th^, 25^th^, 50^th^, 75^th^, and 95^th^) across age cohorts from birth to 2 years. Since sex had no effect on the fiber size in patients 2 years of age or younger, the data from boys and girls were pooled to illustrate myofiber growth across this age range. (B, C) Fiber size by percentile for different age groups from birth to 24 months is also shown separately for males (B) and females (C).

**Figure 5.**
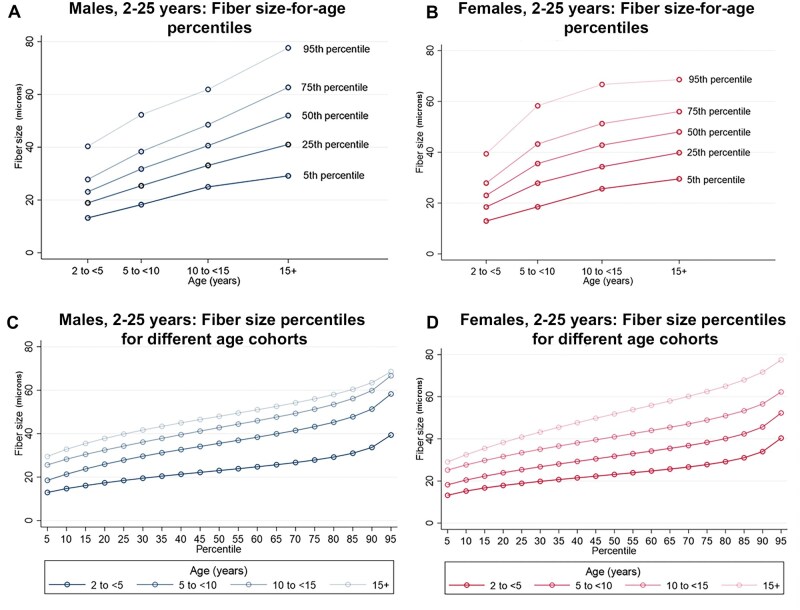
Fiber size growth from 2 to 25 years of age. Assessment of fiber size (minFeret diameter) at select percentiles (5^th^, 25^th^, 50^th^, 75^th^, and 95^th^) across four age groups between 2 and 25 years, shown separately for males (A) and females (B). Fiber size is also shown by percentile and age group in males (C) and females (D) for this older age range.

Patients were separated into 18 age cohorts ranging from 4 weeks to 25 years of age. The age groups, number of biopsies, and biopsy locations are shown in Table 1. Age grouping was selected to account for (1) the very rapid period of myofiber growth within the first year (necessitating multiple groups within the first year), (2) the relatively slow myofiber growth observed after adolescence, and (3) the relative infrequency of diagnostic muscle biopsies in teens and young adults, in comparison to children at younger ages. Following scanning and Visiopharm analysis, the study team reviewed the segmentation masks for all biopsy samples to confirm appropriate segmentation for the vast majority of fibers in a specimen (Figure 2A). After applying manually determined thresholds of reasonable minimum and maximum values for each age cohort to eliminate measured areas of the slide that did not correspond to individual myofibers, results for each patient were graphed as cumulative probability plots to visualize the distribution of data within each biopsy and the comparison of data distribution across patient biopsies taken at the same age (Figure 2B). Summary statistics for biopsies taken from males and females at each age were also calculated (Table 3). With only a few exceptions that may be attributable to small sample sizes for certain age and sex cohorts, fiber size increased with age for both males and females (Figure 3A and B). Calculated percentile data for males and females were also used to construct various growth curves. Due to the rapid period of myofiber growth early in life compared to slower growth after this period, separate curves are provided for the period between birth and 2 years (Figure 3C), and the period between 2 and 25 years (Figure 3D). A demonstration of how fiber size percentiles change over time is shown in Figure 4, with pooled data from male and female patients up to 2 years of age shown in Figure 4A (as there was no impact of patient sex in this age group), and separate male and female data for this age range shown in Figure 4B and C. Fiber size data related to male and female patients 3 years of age and older are shown in shown in Figure 5. More clinically applicable versions of these growth curves are provided in Figures S1 and S2.

**Table 3.** Descriptive statistics by age and sex.

	Females					Males				
Age	N (fibers)	N (biopsies)	Mean	SD	Median	N (fibers)	N (biopsies)	Mean	SD	Median
0-3 months	216 394	9	11.56	5.32	10.87	463 689	12	11.87	7.20	10.51
4-6 months	227 401	5	15.68	6.71	15.23	343 744	9	14.64	7.27	13.71
7-9 months	290 784	6	13.45	5.15	13.04	233 892	7	18.12	10.99	14.65
10-12 months	217 103	6	16.65	8.81	15.30	5913	1	14.47	9.48	12.22
1 year	532 258	11	15.33	7.52	14.26	558 375	14	15.57	8.26	14.75
2 years	431 735	13	20.67	7.83	19.16	445 956	11	22.15	8.38	21.04
3 years	230 798	14	23.48	7.51	23.01	182 853	11	23.45	8.47	22.63
4 years	119 504	9	26.54	13.47	23.81	251 069	10	27.26	11.13	26.74
5 years	50 299	5	36.08	14.03	33.69	104 767	12	29.92	10.56	28.46
6 years	75 203	9	35.00	13.14	32.75	52 527	7	31.42	13.81	28.04
7 years	37 328	3	34.05	12.60	31.64	71 848	8	33.24	11.40	32.27
8 years	44 260	5	34.10	12.85	32.98	100 415	9	30.87	10.23	30.24
9 years	51 771	9	36.33	13.89	35.73	49 938	6	34.88	12.96	33.52
10-12 years	52 807	11	41.91	12.89	40.74	125 969	15	37.80	11.85	36.08
13-15 years	36 294	11	51.83	15.55	51.24	47 736	12	49.18	15.92	47.25
16-18 years	70 900	15	48.22	14.30	46.55	73 267	19	50.84	14.46	50.29
19-21 years	40 037	12	47.49	11.67	46.78	28 637	10	51.88	17.07	50.69
22-25 years	46 639	12	49.54	12.83	49.00	38 120	11	58.19	17.46	58.94

### Fiber size comparisons across sexes

MinFeret diameter data were compared between sexes in each age group, and the related statistics are summarized in Table 4. With only a few exceptions most likely attributable to small sample sizes in certain age groups, there were no significant differences in myofiber size between males and females in the age cohorts at or below the age of 15 years (Figure 3B). In the three age cohorts beyond age 15, however, the males displayed larger fiber sizes in the 75^th^ and 95^th^ percentiles, without a significant difference observed in the 5^th^, 20^th^, or 50^th^ percentiles. This suggests that the smaller fibers in normal biopsy samples do not vary with sex, while the largest fibers are significantly more prominent in males compared to females after 15 years of age.

**Table 4.** Comparing fiber size between males and females in each age group.

Age	Sex	5th percentile		25th percentile		50th percentile		75th percentile		95th percentile	
		Mean ± SD	*P*-value	Mean ± SD	*P*-value	Mean ± SD	*P*-value	Mean ± SD	*P*-value	Mean ± SD	*P*-value
0-3 months	Female	6.09 ± 0.69	0.96	8.66 ± 1.28	0.81	10.77 ± 1.60	0.74	13.15 ± 2.13	0.63	19.54 ± 5.98	0.38
	Male	6.14 ± 0.78		9.19 ± 2.39		11.66 ± 3.01		14.72 ± 3.86		23.46 ± 8.17	
4-6 months	Female	6.41 ± 1.43	0.48	10.08 ± 2.03	0.70	14.07 ± 1.89	0.94	18.23 ± 1.15	0.85	28.46 ± 3.87	0.68
	Male	7.39 ± 1.25		11.15 ± 2.42		23.80 ± 3.19		17.48 ± 4.94		30.76 ± 17.52	
7-9 months	Female	6.68 ± 0.69	0.07	10.53 ± 1.46	0.03	13.78 ± 2.44	0.06	16.84 ± 3.81	0.07	22.55 ± 6.66	0.05
	Male	9.23 ± 2.26		16.61 ± 6.58		20.24 ± 7.59		24.11 ± 9.24		33.52 ± 14.97	
10-12 months	Female	8.64 ± 2.06	0.23	13.58 ± 3.26	0.27	16.36 ± 3.44	0.54	19.68 ± 4.12	0.88	29.98 ± 9.35	0.97
	Male^a^	5.41		7.56		12.22		18.52		30.35	
1 year	Female	6.48 ± 0.84	0.86	12.40 ± 2.75	0.84	16.20 ± 3.67	0.94	19.52 ± 4.24	0.96	28.36 ± 7.06	0.82
	Male	6.66 ± 1.57		11.99 ± 2.87		16.02 ± 4.16		19.67 ± 4.53		29.30 ± 7.47	
2 years	Female	12.68 ± 1.96	0.73	17.18 ± 3.41	0.61	20.62 ± 4.12	0.64	24.61 ± 5.17	0.67	35.18 ± 10.52	0.56
	Male	13.03 ± 1.57		18.22 ± 3.12		21.79 ± 3.64		25.89 ± 3.83		37.06 ± 7.71	
3 years	Female	13.40 ± 2.14	0.99	18.82 ± 2.60	0.91	22.93 ± 2.58	0.83	27.18 ± 2.68	0.91	37.37 ± 5.77	0.55
	Male	13.41 ± 2.36		18.59 ± 3.59		22.38 ± 3.67		26.85 ± 3.21		39.81 ± 9.65	
4 years	Female	12.65 ± 3.28	0.64	19.88 ± 5.59	0.95	26.67 ± 8.28	0.64	33.67 ± 11.11	0.42	48.63 ± 15.10	0.38
	Male	13.20 ± 2.94		20.02 ± 5.09		25.34 ± 5.87		30.94 ± 6.46		44.58 ± 9.98	
5 years	Female	19.01 ± 1.89	0.56	29.23 ± 7.92	0.07	36.49 ± 11.69	0.05	44.34 ± 13.93	0.021	61.80 ± 12.31	0.009
	Male	18.24 ± 2.40		24.32 ± 3.77		29.93 ± 6.60		35.35 ± 8.25		47.58 ± 9.84	
6 years	Female	19.62 ± 2.99	0.57	27.89 ± 4.84	0.46	34.39 ± 6.55	0.49	41.66 ± 9.70	0.96	56.95 ± 13.35	0.99
	Male	18.91 ± 4.26		26.02 ± 6.66		33.56 ± 11.42		41.49 ± 16.96		56.88 ± 21.90	
7 years	Female	18.51 ± 2.64	0.70	28.52 ± 4.32	0.67	36.19 ± 7.62	0.41	42.88 ± 10.21	0.42	56.19 ± 16.05	0.76
	Male	19.17 ± 2.52		27.06 ± 4.36		32.71 ± 4.59		38.94 ± 5.12		54.13 ± 10.75	
8 years	Female	17.32 ± 1.20	0.93	25.14 ± 2.15	0.86	33.26 ± 3.32	0.50	40.56 ± 4.57	0.44	54.83 ± 8.59	0.31
	Male	17.45 ± 1.85		24.65 ± 4.37		30.92 ± 4.45		37.40 ± 4.67		49.08 ± 8.67	
9 years	Female	17.88 ± 2.55	0.73	28.30 ± 8.69	0.32	37.32 ± 9.65	0.21	45.85 ± 10.62	0.24	60.28 ± 11.79	0.77
	Male	17.41 ± 1.19		25.63 ± 3.50		33.25 ± 2.72		41.32 ± 2.13		58.69 ± 4.08	
10-12 years	Female	23.61 ± 2.93	0.59	31.68 ± 4.64	0.67	40.04 ± 5.54	0.47	48.42 ± 6.90	0.41	64.66 ± 9.38	0.22
	Male	23.07 ± 2.82		30.82 ± 5.71		38.82 ± 7.11		46.04 ± 7.68		59.56 ± 9.52	
13-15 years	Female	28.38 ± 2.52	0.20	38.54 ± 6.36	0.84	47.90 ± 6.83	0.68	56.90 ± 7.01	0.54	71.25 ± 6.22	0.44
	Male	29.71 ± 4.86		28.97 ± 8.50		46.82 ± 9.98		55.04 ± 11.21		68.02 ± 12.11	
16-18 years	Female	28.58 ± 1.94	0.62	38.98 ± 5.97	0.18	47.48 ± 7.67	0.026	55.89 ± 9.03	0.014	69.20 ± 11.88	0.07
	Male	29.00 ± 2.33		41.32 ± 4.32		52.26 ± 5.28		62.14 ± 6.69		75.45 ± 7.79	
19-21 years	Female	30.05 ± 2.46	0.16	39.78 ± 4.62	0.90	47.44 ± 6.25	0.11	54.72 ± 7.55	0.007	66.43 ± 8.79	0.002
	Male	28.52 ± 2.47		40.06 ± 7.45		51.66 ± 8.86		63.14 ± 8.68		80.33 ± 8.05	
22-25 years	Female	30.51 ± 3.08	0.25	40.81 ± 5.54	0.67	48.41 ± 6.28	0.05	56.06 ± 7.06	0.001	68.32 ± 7.32	<0.001
	Male	29.30 ± 4.34		41.71 ± 8.05		53.44 ± 8.55		65.93 ± 7.33		82.59 ± 7.46	

a A single biopsy from a male age 10-12 months was available.

### Pilot application of fiber size standards to known disease samples

**Figure 6.**
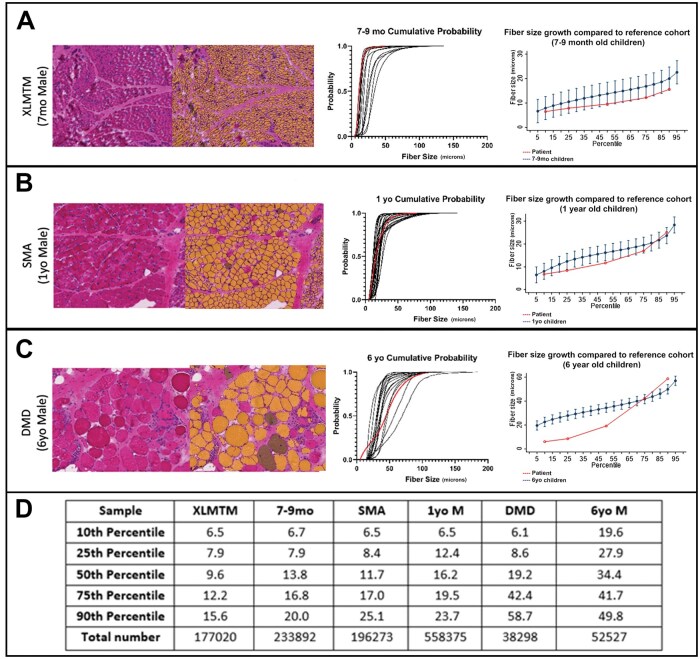
Application of fiber size values to muscle disorders. Biopsies from patients with disorders that affect fiber size were selected to evaluate the practical utility of the fiber size analysis approach used in this study. Disorders tested include X-linked myotubular myopathy (A; XLMTM), spinal muscular atrophy (B; SMA), and Duchenne muscular dystrophy (C; DMD). The left panels display representative microscopic images and analysis masks from each biopsy (where areas recognized as fibers are shown in yellow or blue). The middle panels display the cumulative probability plots related to the measurements in each biopsy (red) compared to the individual cumulative probability plots from the matching normal muscle biopsy age cohort. The right panels display the fiber size vs percentile plot for each biopsy specimen (red) compared to the data for the matched age cohort, with 95% confidence intervals shown. (D) Individual key percentiles for each disease specimen in comparison to the age-matched normal group in the adjacent column are shown; data are fiber size (minFeret diameter) values in microns (μm). Abbreviations: M, male; mo, months old; yo, years old.

To demonstrate how automated fiber size measurements, cumulative probability plots, and growth curves can be used in an evaluative setting, a pilot study was performed to assess clinical biopsies with fiber size abnormalities and compare the measurements from those biopsies with the normal fiber size ranges developed from our data. The selected cases are shown in Figure 6 and Figure S3. Histological abnormalities corresponded to different aberrations from the normal size distributions for the same age range and sex. For instance, an X-linked myotubular myopathy case from a 7-month-old boy showed a normal fiber size distribution, but all fibers were very small (XLMTM; Figure 6A). Despite being small, there was overlap with some histologically normal cases from this age group based on the cumulative probability plot. In contrast, cases of spinal muscular atrophy (SMA; Figure 6B) and Duchenne muscular dystrophy (DMD; Figure 6C) showed fiber sizes that were within the range of normal for age and sex, but with a much wider size distribution due to excessive fiber size variation. This study did not have the opportunity to track serial muscle biopsies in individual patients over time but this would likely be useful for studies related to growth, disease progression, or treatment efficacy when studying factors that are likely to impact muscle fiber size.

## DISCUSSION

This study represents the latest effort to establish standards for myofiber size in pediatric and young adult patient populations. Early publications in this area were limited in scope due to the restriction to manual myofiber measurements.^1–6^ Using these manual approaches, it was possible to establish standards for adult patients because normal fiber size is stable after the teenage years.^6^ However, establishing accurate fiber size standards in childhood requires the evaluation of a much larger set of biopsy specimens across a broad range of pediatric ages. The development of automated image analysis platforms has recently made such studies feasible.

A recent study by Evangelista et al. used image analysis to perform myofiber size and type measurements in 82 deltoid muscle biopsies from patients aged 0 to 18 years.^7^ In addition to providing standard fiber size data for the deltoid, that study established the impact of age on fiber type proportions. In contrast, our study focused solely on the fiber size measurements but had a much larger sample size, included other commonly sampled anatomic sites (with the preponderance of the vastus lateralis/quadriceps/thigh region biopsies), assessed a larger number of fibers per biopsy, and provided greater granularity at the ages of 0-2 years (when the rate of myofiber growth is high and when many diagnostic biopsies are done). Therefore, these two papers provide complementary information on an important knowledge gap in muscle biopsy diagnostics. While a direct comparison is somewhat difficult given the different ways the data are presented in the two studies, the minFeret diameters reported in both papers appear very similar, based on the key percentiles of interest. For instance, the 95^th^ percentile of deltoid myofiber size in females in the Evangelista et al study was approximately 70 microns,^7^ whereas in the current study, the range of sizes observed in 16-18-year-old females was 69.20 ± 11.88 microns. Similarly, the 95^th^ percentile of deltoid myofiber size in males in the Evangelista et al study was approximately 85 microns, whereas in the current study, the range of sizes observed in 16-18-year-old males was 75.45 ± 7.79 microns. Interestingly, Evangelista et al identified a plateau in fiber size at approximately 11 years of age for females and 16 years of age for males with some size differences observed in the plots of male and female minFeret diameter data before the age of 10 years.^7^ In our study, differences between fiber size in male and female biopsy samples were not evident prior to 15 years of age, concordant with the findings in the two historic autopsy studies, which reported sex differences in fiber size emerging around age 15.^2^^,^^3^ Possible explanations for these small differences include differences in the approach to age grouping, differences in the number of biopsy samples available for study at each of these ages, and differences in the anatomic location that was sampled (deltoid vs quadriceps femoris). Interestingly, Oertel found that these post-pubertal sex differences in fiber size were due to the differential growth of type 2 fibers: in males, type 2 fibers grew more and often became larger than type 1 fibers by age 20, whereas in females, type 2 fibers remained smaller than type 1 fibers.^3^

It is well-established that muscle growth in postnatal, non-injured muscle occurs through hypertrophy (growth of myofiber size) rather than hyperplasia (increasing the number of myofibers). As a result, one would expect that the myofiber growth patterns would mimic the patterns of body size growth in children, including bursts of growth coinciding with periods of rapid growth around puberty. However, our dataset demonstrated a rapid increase in myofiber size in the first year of life followed by a progressive slowing of muscle growth after that age. While the observed differences in myofiber size between males and females after 15 years of age are likely related to hormonal influences, we did not detect a period of rapid myofiber diameter growth at typical pubertal ages in males or females. It is likely that through crosstalk between growth in bone length and longitudinal muscle stretching (another mechanism of muscle growth induction), muscle growth during puberty occurs primarily in the longitudinal aspect of myofibers and the minFeret diameter measurements may not be sensitive indicators of muscle growth during these periods.

Quantifying myofiber size is an increasingly important aspect of studies on muscle disease. In some muscle disorders, abnormally small myofibers are a potential cause of weakness, and the degree of fiber size abnormality can correlate with functional deficits in experimental animals or human patients.^10–12^ The pilot data collected in this study also demonstrate how the patterns of myofiber abnormality can be different in different disease states. However, quantifying myofiber size can be challenging and time-consuming, as an appropriate sample may constitute thousands of fibers, and the diameter for measurement may depend on fiber orientation. Thus, while muscle fiber size measurements can be helpful in research and clinical settings, they may be prohibitively time-consuming to manually perform on sufficient numbers of fibers to yield accurate, meaningful results. As such, algorithms for automated fiber size measurement are warranted.

Advances in scanning technology and the increasing accessibility of AI image analysis software have greatly improved the field’s capacity to provide quantitative datasets related to myofiber size and other pathological abnormalities. However, it is essential to consider that automated software analysis is only as good as the evaluated material. This study employed automated measurement techniques and manual post-analysis for quality control, along with several advanced statistical approaches. Care was taken during biopsy selection to exclude samples that: (1) displayed any significant histological abnormalities, (2) displayed significant freezing or other preparation artifacts, and (3) displayed significant proportions of non-muscle tissue. Following data generation, it was necessary to cross-check the “mask” images from each sample to ensure that myofiber segmentation was appropriate compared to the visual impression. Limited manual measurements of the largest and smallest myofibers in each age group were performed to establish realistic minimum and maximum fiber sizes; this excluded measured structures that were too small or too large to be myofibers. While non-myofiber structures within the allowed size range were not excluded using this approach, our work comparing manual to automated assessments indicates that their inclusion does not impact the overall dataset due to the large number of myofibers measured under these conditions.

A comparison of myofiber size in male and female children identified that myofibers in both sexes are similar in size throughout most of childhood. Starting at age 15 years, myofiber size in males becomes increasingly different from that of females due to an increase in size in the largest fiber populations (primarily observed in the 50^th^, 75^th^, and 95^th^ percentile diameters at these ages). The survey of deltoid biopsies by Evangelista et al found differences in fiber size measurements between males and females after age 11.^7^ Jansson et al. studied muscle biopsies from 69 males and 47 females at 16 years of age and found that muscle fiber areas for all fiber types were larger in males than in females.^5^ While the specific age at which myofiber sizes diverge between males and females may depend on the muscle evaluated or the study population, all these studies are consistent with a pattern of growth that is unaffected by patient sex until late childhood, with higher myofiber sizes in males beyond 11-14 years of age.

A significant limitation of this study is that we collected the fiber size data from “normal-appearing” biopsies rather than biopsies obtained from healthy patients without clinical symptoms of muscle disease; it is possible that some of the samples we included in our dataset came from functionally abnormal muscles that were normal in appearance. A prospective study of muscle samples from healthy human subjects would have addressed this limitation but such a study cannot be performed in children due to ethical reasons, especially given the need to cover such a large range of patients of both sexes across the continuum of childhood and early adulthood. While autopsy cases are a potential source of muscle tissue from patients without known neuromuscular disease, pediatric autopsies are rare and often confounded by long hospitalizations prior to death, which can lead to acquired muscle abnormalities (disuse atrophy or critical illness myopathy). In addition, routine autopsy tissue collection typically does not include limb muscles, nor is the sampled muscle tissue frozen and stained in the same way as diagnostic muscle biopsies. Therefore, we decided that the most practical approach to obtain a sufficiently large number of cases across the desired age range was to focus on a retrospective review of muscle biopsy reports to identify cases without significant pathological abnormalities and then to specifically reassess these cases for suitability for this study. This approach focusing on histologically normal cases also addressed the issue of inconsistent clinical data in the original biopsy reports, as there was no way during this retrospective review to confirm that the initial, and often limited, clinical suspicion/history proved to be the correct final diagnosis. Reassuringly, however, our findings are highly concordant with the results of the two autopsy-based studies which, while significantly smaller in scope, also investigated muscle fiber growth over the pediatric age range continuum.^2^^,^^3^ In particular, the study performed by Oertel,^3^ which used frozen deltoid and quadriceps autopsy samples for analysis and grouped patients into similar age-based cohorts as we did, reported that the mean diameter of the muscle fibers was 10-12 microns shortly after birth and 40-60 microns at age 15-20 years.

Given our approach of using only H&E-stained slides from archival biopsy samples, some important questions remain to be answered by future studies. The range of standard fiber size is different in diverse muscle groups,^13^ and it would be beneficial to perform a similar study focused on a subset of different muscles at a specific age. While we included all histologically normal biopsies at each institution that fit our inclusion criteria, there were insufficient biopsy specimens outside the vastus lateralis/quadriceps muscles to allow for cross-muscle comparisons. Given that fiber type cannot be accurately assessed using H&E-stained slides, the current study provides no information about the changes in the fiber type composition in the quadriceps muscle with increasing age or between males or females of the same age. Evangelista et al performed fiber-type assessments in their study of pediatric deltoid muscles and observed approximately 50% type 1 fibers up to 11 ± 3 years of age, after which the proportion of type 1 fibers dropped to approximately 40%.^7^ On the other hand, Oertel reported that the deltoid and quadriceps muscles of children shortly after birth contained about 40% of type 1 fibers, with the type 1 fiber percentage increasing to about 60% within the first 2 postnatal years and then remaining relatively constant.^3^ Our anecdotal experience with diagnostic biopsies from the quadriceps/vastus lateralis/thigh is consistent with these observations, as normal pediatric muscle biopsies often have approximately 50% type 1 fibers. Ideally, a comprehensive study of fiber type distributions at different ages and from different muscles could be performed to address these questions. We hope that the approach developed and described in our study enables future studies such as these to more efficiently incorporate data from large, diverse cohorts of pediatric and young adult patients.

## Supplementary Material

nlaf123_Supplementary_Data
