# Supplementary material for: Establishment of normal myofiber size distribution in children and young adults
Source: J Neuropathol Exp Neurol. 2025 Nov 4;84(12):1159–73. doi: 10.1093/jnen/nlaf123 (PMC12713552; doi:10.1093/jnen/nlaf123)
Supplement: nlaf123_Supplementary_Data [file nlaf123_supplementary_data.zip › Table S1.docx]

**Table S1. Ranges of fiber size diameter analyzed for each age range**Fiber diameters below the minimum or above the maximum were excluded from cumulative probability and frequency histogram calculations and plots.

| **Age range** | **Minimum diameter (µm)** | **Maximum diameter (µm)** |
| --- | --- | --- |
| 0-12 months | 5 | 200 |
| 2-4 years | 10 | 200 |
| 5-9 years | 15 | 200 |
| - 1. years | 20 | 200 |
| >13 years | 25 | 200 |
