# Supplementary figures and images for: Establishment of normal myofiber size distribution in children and young adults
Source: J Neuropathol Exp Neurol. 2025 Nov 4;84(12):1159–73. doi: 10.1093/jnen/nlaf123 (PMC12713552; doi:10.1093/jnen/nlaf123)

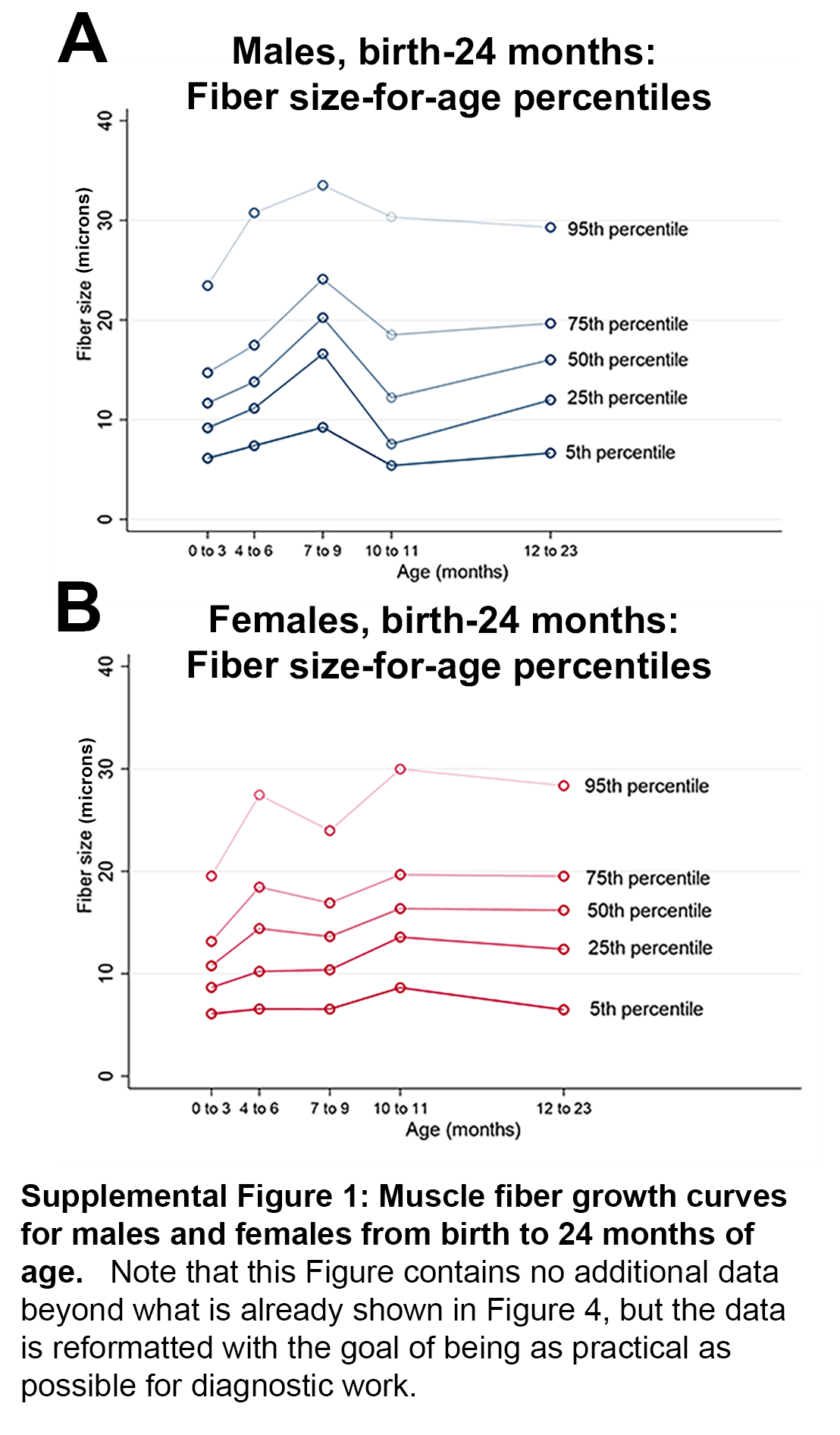

Supplement: nlaf123_Supplementary_Data [file nlaf123_supplementary_data.zip › Sup Figure 1.tif]

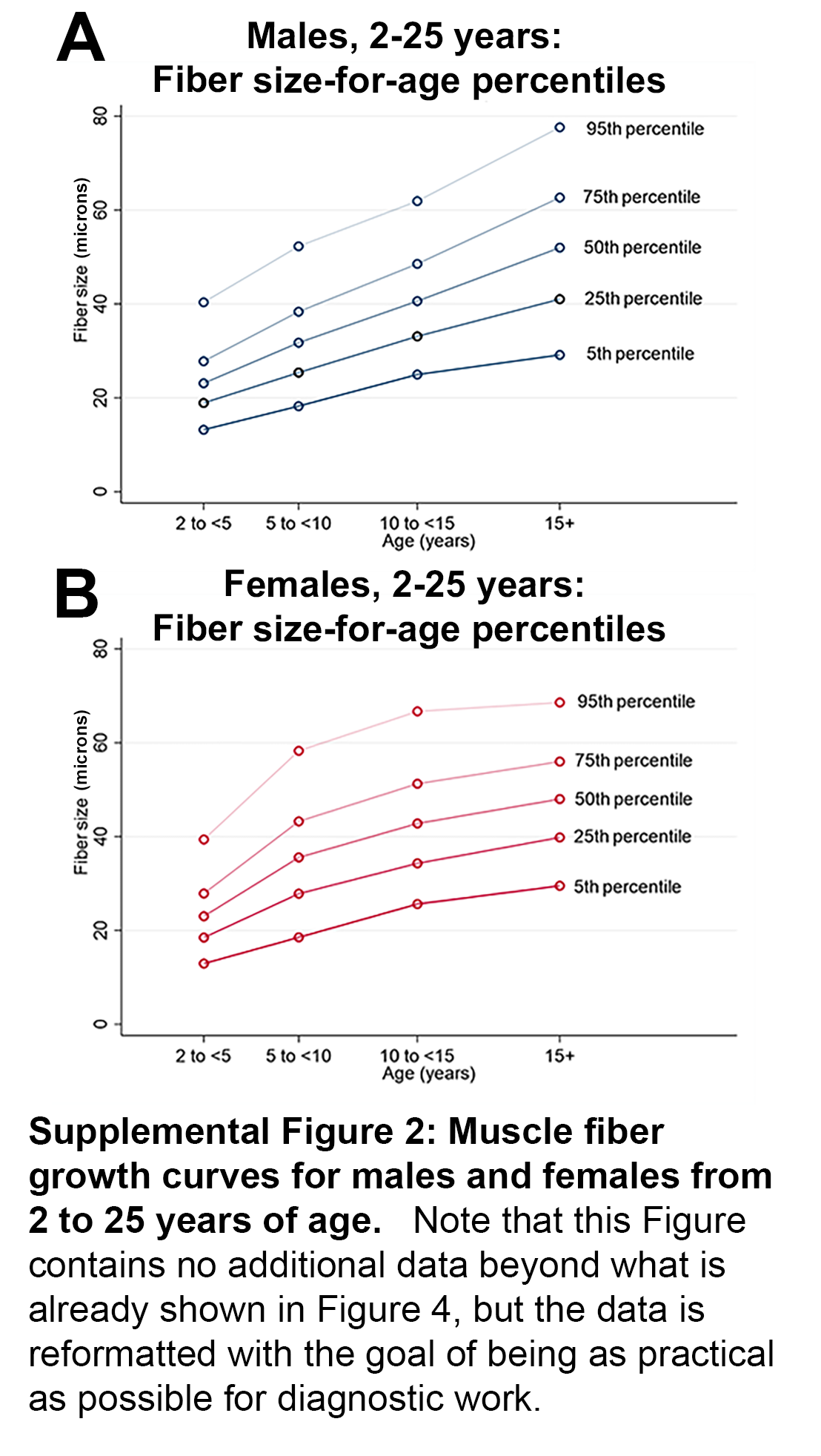

Supplement: nlaf123_Supplementary_Data [file nlaf123_supplementary_data.zip › Sup Figure 2.tif]

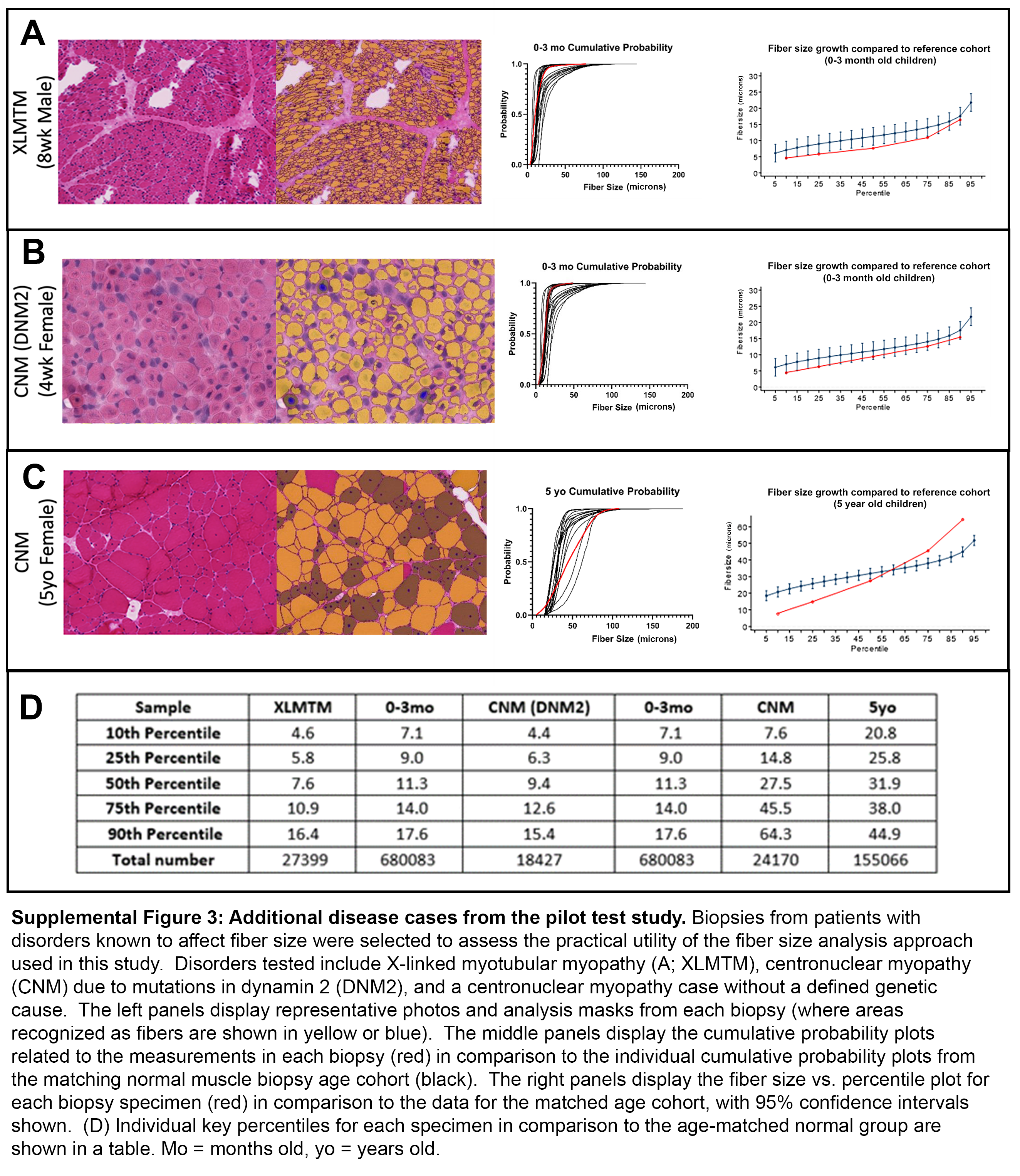

Supplement: nlaf123_Supplementary_Data [file nlaf123_supplementary_data.zip › Sup Figure 3.tif]
